# Miliary TB and COVID-19 Coinfection in a Patient With a History of Post-polycythemia Vera Myelofibrosis Treated With Ruxolitinib: A Case Report

**DOI:** 10.7759/cureus.63791

**Published:** 2024-07-03

**Authors:** Maria Loutsou, Vasiliki E Georgakopoulou, Nikolaos Roussakis, Konstantina Chadia, Paschalis Steiropoulos

**Affiliations:** 1 Department of Respiratory Medicine, Medical School, Democritus University of Thrace, Alexandroupolis, GRC; 2 Department of Pathophysiology/Pulmonology, Laiko General Hospital, Athens, GRC

**Keywords:** post-polycythemia vera myelofibrosis, immunosuppression, ruxolitinib, covid-19 coinfection, miliary tuberculosis

## Abstract

The coronavirus disease 2019 (COVID-19) pandemic has significantly impacted the diagnosis and management of tuberculosis (TB), a major public health issue. This case report discusses a 70-year-old female with post-polycythemia vera myelofibrosis (post-PV MF) treated with ruxolitinib who developed miliary TB amidst a COVID-19 infection. The patient presented with a flu-like syndrome over the past week with fatigue and weight loss the last month. When she was admitted to the hospital, the real-time polymerase chain reaction (RT-PCR) for severe acute respiratory syndrome coronavirus 2 (SARS-CoV-2) was positive. Despite the typical COVID-19 presentation, her clinical and radiographic features raised suspicion for disseminated TB. Diagnostic tests, including bronchoscopy and PCR for *Mycobacterium tuberculosis*, confirmed miliary TB. She was treated with a standard antitubercular regimen, leading to symptomatic improvement. The interplay between COVID-19 and TB is complex, with COVID-19-induced immunosuppression, particularly lymphocytopenia, facilitating TB reactivation. Additionally, ruxolitinib, a Janus kinase (JAK) inhibitor used for myelofibrosis, impairs immune defense mechanisms, increasing infection risk, including TB. Prompt and accurate diagnosis of TB in the context of COVID-19 is crucial for effective management and improved patient outcomes. Clinicians should remain vigilant for TB reactivation in patients undergoing treatments such as ruxolitinib and consider alternative diagnoses despite positive SARS-CoV-2 tests. This report highlights the necessity for a comprehensive evaluation and timely intervention to mitigate the compounded risks of COVID-19 and TB.

## Introduction

The global health landscape has dramatically changed with the emergence of the coronavirus disease 2019 (COVID-19) pandemic, which had profound implications for the management and diagnosis of other infectious diseases, notably tuberculosis (TB). TB remains a significant public health issue worldwide. Approximately 10.6 million people were affected by the disease in 2021, reflecting a 4.5% increase from the previous year, largely due to the disruptions caused by the pandemic [1]. In Greece, the incidence of TB was reported to be two cases per 100,000 people in 2022, indicating the persistent presence of this disease despite the overall improvements in healthcare [2].

Studies have shown that COVID-19 patients often exhibit significant lymphocytopenia, particularly a decrease in CD4+ and CD8+ T lymphocytes, which are crucial for mounting an effective immune response against TB [3]. This immunosuppression can facilitate the reactivation of latent TB infections (LTBI), leading to more severe and disseminated forms of the disease, such as miliary TB, which accounts for 1%-2% of all TB cases and up to 20% of extrapulmonary TB cases [4].

The intersection of TB and COVID-19 is further complicated by the use of immunosuppressive therapies such as ruxolitinib, which is a medication used in the treatment of myeloproliferative disorders. Ruxolitinib, a Janus kinase (JAK) inhibitor, impairs cytokine signaling pathways essential for immune defense, thereby increasing the risk of infections, including TB [5]. Reports have documented cases of ruxolitinib-associated reactivation of LTBI, emphasizing the need for thorough screening and vigilant monitoring in patients receiving this therapy [6].

In this case report, we present a 70-year-old female with a history of post-polycythemia vera myelofibrosis (post-PV MF) treated with ruxolitinib who developed miliary TB in the context of the COVID-19 infection. This case underscores the critical importance of considering alternative diagnoses in patients presenting with atypical clinical and radiographic features during the course of COVID-19, highlighting the necessity for prompt and accurate diagnosis to ensure appropriate management and improve patient outcomes.

## Case presentation

A 70-year-old female with a history of post-PV MF, treated with ruxolitinib since 2021, presented to a tertiary care hospital in North Greece. She reported experiencing fatigue, malaise, and a weight loss of approximately 7-8 kg during the last month, along with a loss of appetite over the past month, followed by flu-like symptoms for the past week. Additionally, she had an evening rise in temperature, peaking at 38°C, accompanied by night sweats. There were no reported difficulties with swallowing or breathing, headaches, or bowel or bladder disturbances.

Her medical history included hypertension and diabetes mellitus, under regular treatment. There was no history of human immunodeficiency virus (HIV) infection or contact with TB within her family or community. She had not received the COVID-19 vaccine and had no history of COVID-19 infection. The patient was diagnosed with polycythemia vera in 2019 and was treated with hydroxyurea until 2021. Then, she was conducted in a bone marrow biopsy due to the presence of splenomegaly and hydroxyurea resistance. The biopsy results revealed the diagnosis of post-PV MF. Her treatment was amended to ruxolitinib at a dose of 10 mg twice daily, as the preferred medication used in these cases.

Her rapid antigen tests for COVID-19 and flu (type A and type B) were negative. On examination, her oxygen saturation was 98% (FiO2: 21%), blood pressure was 140/90 mmHg, heart rate was 120 bpm, temperature was 38.3°C, and respiratory rate was 15 breaths per minute. Cardiopulmonary and neurological examinations were normal, with no signs of meningitis. There was no evidence of pallor, rashes, icterus, cyanosis, clubbing, lymphadenopathy, or pedal edema. The abdomen was soft, with no hepatomegaly but with splenomegaly.

Laboratory investigations revealed lymphocytopenia and anemia. The inflammatory markers C-reactive protein (CRP) and erythrocyte sedimentation rate (ESR) were elevated. Liver function tests indicated mild impairment (Table 1).

**Table 1:** Laboratory investigations on admission HCT: hematocrit, Hb: hemoglobin, CRP: C-reactive protein, ESR: erythrocyte sedimentation rate, SGOT: serum glutamic-oxaloacetic transaminase, SGPT: serum glutamic pyruvic transaminase, ALP: alkaline phosphatase, GGT: gamma-glutamyl transferase, LDH: lactate dehydrogenase

Parameter	Value	Reference range
Lymphocytes	0.42 × 10³/μL	1-3 × 10³/μL
HCT	32.1%	36%-47%
Hb	10.9 mg/dL	12-15 mg/dL
CRP	7.61 mg/dL	<1 mg/dL
ESR	90 mm/hour	0-20 mm/hour
SGOT	50 IU/L	0-35 IU/L
SGPT	43 IU/L	0-35 IU/L
ALP	159 IU/L	44-147 IU/L
GGT	70 IU/L	0-30 IU/L
Total bilirubin	1.54 mg/dL	0.1-1.2 mg/dL
LDH	520 IU/L	120-230 IU/L

**Figure 1:**
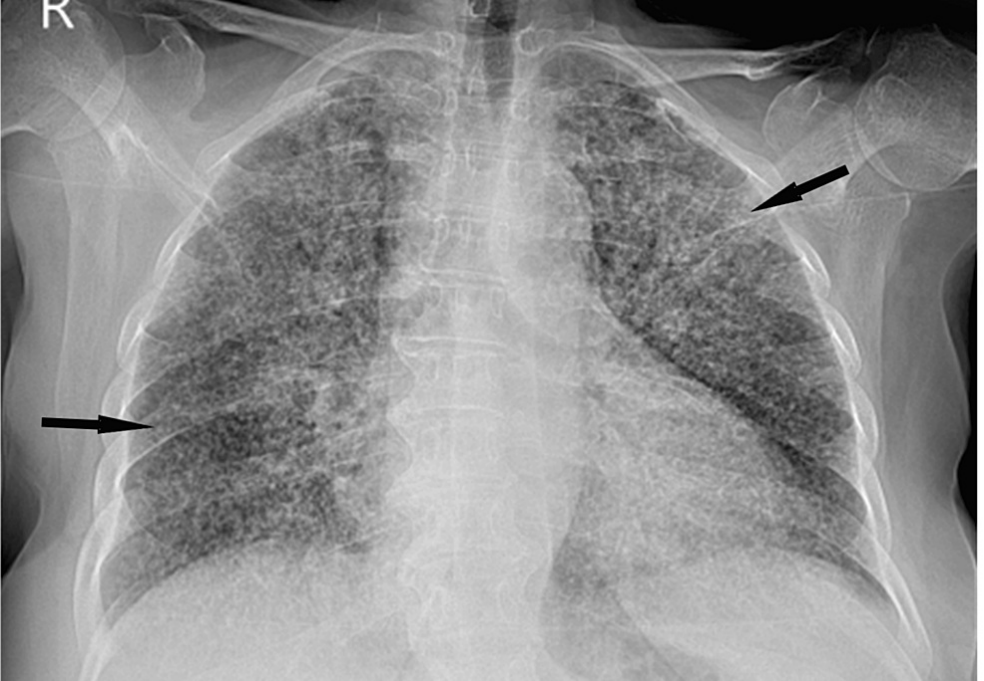
Chest X-ray on admission Arrows show diffusely distributed infiltrates with micronodular patterns.

Urinalysis was normal, and urine culture was negative. Tests for HIV, hepatitis B virus (HBV), and hepatitis C virus (HCV) were negative. The reverse transcription-polymerase chain reaction (RT-PCR) test for severe acute respiratory syndrome coronavirus 2 (SARS-CoV-2) returned positive after 24 hours. Her chest X-ray showed diffusely distributed reticulonodular infiltration (Figure 1).

**Figure 2:**
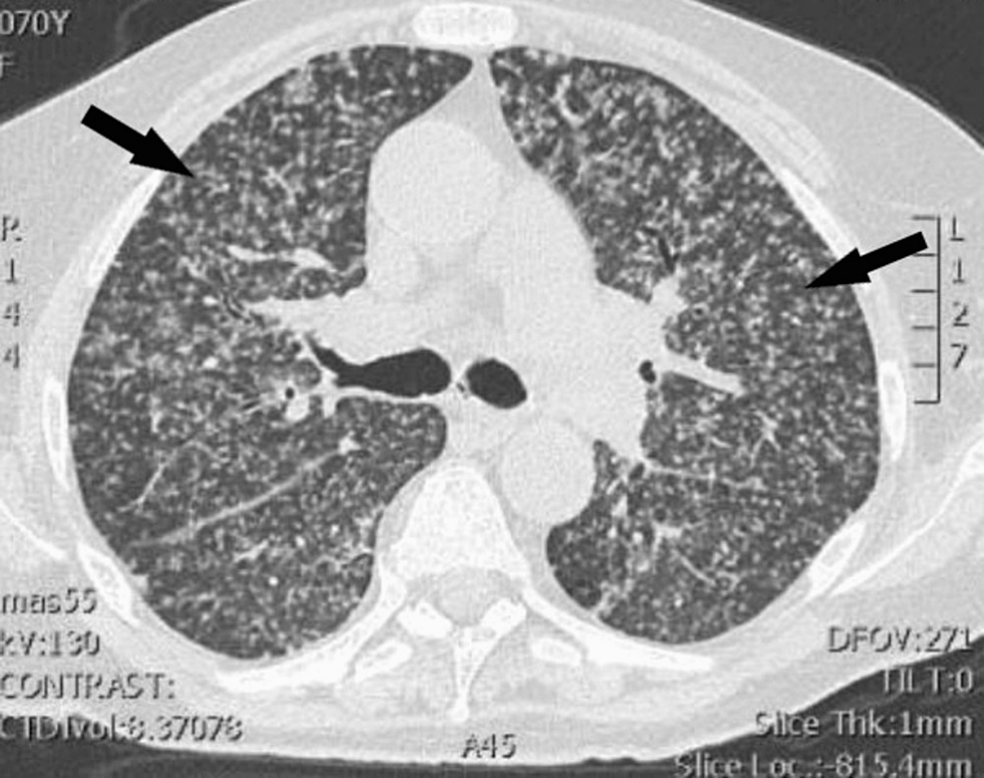
Chest computed tomography without contrast (lung window) Arrows show diffuse bilateral and widespread miliary nodules.

The chest computed tomography (CT) scan (without contrast) revealed diffuse bilateral miliary nodules in all lung segments (Figure 2).

The differential diagnosis included COVID-19 pneumonia and miliary TB.

The patient was treated with remdesivir and a prophylactic dose of low-molecular-weight heparin according to COVID-19 treatment guidelines, as she was immunocompromised and at high risk of developing severe COVID-19 disease. However, there was high suspicion of disseminated TB according to her medical history and chest X-ray and CT scan findings.

From the onset of the patient's presentation, we commenced investigations for possible active TB. The tuberculin skin test (TST) was conducted by injecting 0.1 mL of tuberculin-purified protein derivative (PPD) into the inner surface of the forearm. After 72 hours, the result was 0 mm, which could be a false negative due to the patient's history of post-PV MF or a recent TB infection within the past 8-10 weeks. A blood sample was then analyzed using the QuantiFERON test, yielding an indeterminate result, thus rendering the likelihood of *Mycobacterium tuberculosis* infection inconclusive.

To enhance the sensitivity of our diagnostic approach and aid in differential diagnosis, we performed bronchoscopy with the collection of bronchoalveolar lavage (BAL). The BAL specimen was subjected to acid-fast bacilli staining, gene Xpert PCR, and cultures for TB, as well as common bacteria and fungi. The PCR test confirmed the presence of *Mycobacterium tuberculosis*. Consequently, the patient was initiated on a four-drug antitubercular regimen comprising rifampin, isoniazid, pyrazinamide, and ethambutol, in accordance with national TB treatment guidelines. Ruxolitinib was discontinued. The decision to discontinue ruxolitinib was made to reduce immunosuppression and allow the patient's immune system to combat the tuberculosis infection more effectively. This approach is crucial in managing TB in immunocompromised patients, where the balance between treatment efficacy and infection control must be carefully maintained. The patient exhibited symptomatic improvement within one week of initiating the antitubercular therapy.

## Discussion

TB remains a major public health issue worldwide. In 2021, the number of people who were affected by TB, including drug-resistant TB, increased after many years of gradual decline, a setback attributed to the COVID-19 pandemic's effect [7].

Risk factors for TB include immunosuppressive conditions (such as HIV) or drugs, comorbidities (diabetes, chronic renal or hepatic failure, chronic obstructive disease, and lymphoproliferative disorders), young age, and socioeconomic and behavioral factors such as tobacco smoke, alcohol or drug use, and poor living conditions (e.g., overcrowded households, homelessness, malnutrition, low accessibility to healthcare services, and poor personal hygiene) [8]. The patient had a history of diabetes mellitus, and this is also a risk factor for TB in our case. In the last few years, epidemiology data on miliary TB have dramatically changed because of the widespread use of biological agents, chemotherapy, and immunosuppressant drugs [4].

COVID-19 can dysregulate the immune system, which could predispose a patient to reactivation of LTBI. CD4+ and CD8+ T lymphocytes were significantly decreased in patients with COVID-19, and this had an impact both on the severity of the disease and on the prognosis. CD4+ T cells are key components of the immune response against intracellular infections, including TB infection [9]. There are cases in the literature with COVID-19 and tuberculosis coinfection [10]. During latent TB infection, the persistence of mycobacteria induces a chronic pro-inflammatory response within the lung parenchyma, which is important to maintain the structural integrity of the granuloma [11]. Tumor necrosis factor (TNF) and INF-γ are the main cytokines that contribute to the formation of granulomas, and these cytokines simultaneously play a key role in the pro-inflammatory immunomodulation of the response against SARS-CoV-2 [12].

Another possible hypothesis for the reactivation of LTBI in our patient is the treatment with ruxolitinib. The suggested mechanism of action of ruxolitinib is the attenuation of cytokine signaling via the inhibition of JAK1 and JAK2, resulting in antiproliferative and proapoptotic effects [13]. Ruxolitinib is authorized for the treatment of disease-related splenomegaly or symptoms in adult patients with primary myelofibrosis (MF) (also known as chronic idiopathic MF), post-PV MF, or post-essential thrombocythemia MF. This therapeutic agent is indicated for the treatment of adult patients with PV who are resistant to or intolerant of hydroxyurea. Currently, ruxolitinib is also indicated for the treatment of patients with graft-versus-host disease (GvHD) [14].

The pathophysiological mechanism that explains the relationship between this therapeutic regimen and the reactivation of TB is the disruption of JAK-STAT signaling. This disruption affects immune homeostasis and impairs the maturation and function of dendritic and T cells. The main side effect of inhibitors of JAK1 and JAK2 can be an increased risk of infection, related to a depressed Th1 response and a reduced production of interferon-gamma (INF-γ). IFN-γ is a key cytokine involved in protective immunity against *Mycobacterium tuberculosis*, regulating the expression of genes involved in antimycobacterial effector functions [15]. There are several case reports that present the ruxolitinib-associated reactivation of LTBI [13,16-20]. The diagnosis of latent TB with PPD and the QuantiFERON test may be misleading in patients after the initiation of ruxolitinib. Moreover, PPD shows anergy in miliary TB and can become positive after the initiation of antituberculosis treatment. So, it explains why clinicians have to screen for latent TB prior to starting therapy with ruxolitinib.

## Conclusions

In conclusion, this case report underscores the critical interplay between COVID-19 and miliary tuberculosis TB, particularly in immunocompromised patients undergoing treatment with ruxolitinib. The dual diagnosis of COVID-19 and miliary TB in a patient with post-polycythemia vera myelofibrosis emphasizes the necessity for clinicians to maintain a high index of suspicion for TB, even when a positive COVID-19 test might suggest a straightforward viral pneumonia diagnosis. Prompt and accurate diagnosis of TB, alongside the management of COVID-19, is crucial for improving patient outcomes. This case illustrates the complexities in diagnosing and treating coinfections and highlights the need for comprehensive patient evaluations to ensure timely and effective therapeutic interventions. The findings advocate for vigilant monitoring and preemptive screening for latent TB in patients receiving immunosuppressive therapies such as ruxolitinib to mitigate the risks of reactivation and severe disease progression.

## References

[1] Falzon D, Zignol M, Bastard M, Floyd K, Kasaeva T (2023). The impact of the COVID-19 pandemic on the global tuberculosis epidemic. Front Immunol.

[2] (2024). Incidence of tuberculosis (per 100,000 people). https://data.worldbank.org/indicator/SH.TBS.INCD.

[3] Qin C, Zhou L, Hu Z (2020). Dysregulation of immune response in patients with coronavirus 2019 (COVID-19) in Wuhan, China. Clin Infect Dis.

[4] Sharma SK, Mohan A, Sharma A (2016). Miliary tuberculosis: a new look at an old foe. J Clin Tuberc Other Mycobact Dis.

[5] Elli EM, Baratè C, Mendicino F, Palandri F, Palumbo GA (2019). Mechanisms underlying the anti-inflammatory and immunosuppressive activity of ruxolitinib. Front Oncol.

[6] Abidi MZ, Haque J, Varma P, Olteanu H, Guru Murthy GS, Dhakal B, Hari P (2016). Reactivation of pulmonary tuberculosis following treatment of myelofibrosis with ruxolitinib. Case Rep Hematol.

[7] Stephenson J (2022). WHO report: years of progress in global tuberculosis upset by COVID-19 pandemic. JAMA Health Forum.

[8] Narasimhan P, Wood J, Macintyre CR, Mathai D (2013). Risk factors for tuberculosis. Pulm Med.

[9] Liu Z, Long W, Tu M (2020). Lymphocyte subset (CD4+, CD8+) counts reflect the severity of infection and predict the clinical outcomes in patients with COVID-19. J Infect.

[10] Tham SM, Lim WY, Lee CK (2020). Four patients with COVID-19 and tuberculosis, Singapore, April-May 2020. Emerg Infect Dis.

[11] Kampmann B, Hemingway C, Stephens A (2005). Acquired predisposition to mycobacterial disease due to autoantibodies to IFN-gamma. J Clin Invest.

[12] Ostojic A, Vrhovac R, Verstovsek S (2012). Ruxolitinib for the treatment of myelofibrosis: its clinical potential. Ther Clin Risk Manag.

[13] Colomba C, Rubino R, Siracusa L, Lalicata F, Trizzino M, Titone L, Tolomeo M (2012). Disseminated tuberculosis in a patient treated with a JAK2 selective inhibitor: a case report. BMC Res Notes.

[14] Kiladjian JJ, Winton EF, Talpaz M, Verstovsek S (2015). Ruxolitinib for the treatment of patients with polycythemia vera. Expert Rev Hematol.

[15] Khalid F, Damlaj M, AlZahrani M, Abuelgasim KA, Gmati GE (2021). Reactivation of tuberculosis following ruxolitinib therapy for primary myelofibrosis: case series and literature review. Hematol Oncol Stem Cell Ther.

[16] Hopman RK, Lawrence SJ, Oh ST (2014). Disseminated tuberculosis associated with ruxolitinib. Leukemia.

[17] Branco B, Metsu D, Dutertre M, Marchou B, Delobel P, Recher C, Martin-Blondel G (2016). Use of rifampin for treatment of disseminated tuberculosis in a patient with primary myelofibrosis on ruxolitinib. Ann Hematol.

[18] Chen YH, Lee CH, Pei SN (2015). Pulmonary tuberculosis reactivation following ruxolitinib treatment in a patient with primary myelofibrosis. Leuk Lymphoma.

[19] Keizer S, Gerritsen R, Jauw Y, Janssen J, Koopman B, Bresser P (2015). [Fatal tuberculosis during treatment with ruxolitinib]. Ned Tijdschr Geneeskd.

[20] Palandri F, Polverelli N, Catani L, Vianelli N (2015). Ruxolitinib-associated tuberculosis: a case of successful ruxolitinib rechallenge. Ann Hematol.

